# Epidermal Inclusion Cyst of the Clitoris: A Case Report

**DOI:** 10.7759/cureus.29066

**Published:** 2022-09-12

**Authors:** Indira Prasad, Sudwita Sinha, Shreekant Bharti, Jyoti Singh, Simran Dureja

**Affiliations:** 1 Obstetrics and Gynecology, All India Institute of Medical Sciences, Patna, Patna, IND; 2 Pathology, All India Institute of Medical Sciences, Patna, Patna, IND

**Keywords:** benign cyst of clitoris, epidermal cyst, inclusion cyst, epidermal inclusion cyst, spontaneous cyst of clitoris

## Abstract

Epidermal inclusion cysts are cysts occurring in intradermal or subcutaneous regions with the cyst wall consisting of the true epidermis. They are slow-growing tumors that occur most commonly on the scalp, neck, face, and trunk. They are common in infancy and childhood or after female external genitalia mutilation. Herein, we report a case of a spontaneously occurring epidermal inclusion cyst of the clitoris, a rare location in a 43-year-old woman which was removed by local excision.

## Introduction

Epidermal cysts are usually solitary, asymptomatic, slow-growing tumors that are cystic, round, and elevated [1]. They occur most commonly on the scalp, neck, face, and trunk although external genitalia may also be affected and may include cysts of the scrotal, labial, or clitoral region [1-3]. They can arise spontaneously or due to traumatic implantation of the epidermis into the dermal or subcuticular region [1]. The cyst wall is composed of squamous, granular, and horn cells seen in the true epidermis and the cyst wall is filled with laminated layers of horny material [1]. A variety of benign and malignant tumors can involve the clitoris. However, an epidermal inclusion cyst of the clitoris without a history of trauma is an uncommon entity with only a few cases reported in the literature to date. The prognosis after surgical treatment is usually good with no recurrence or malignant transformation reported till date.

## Case presentation

**Figure 1:**
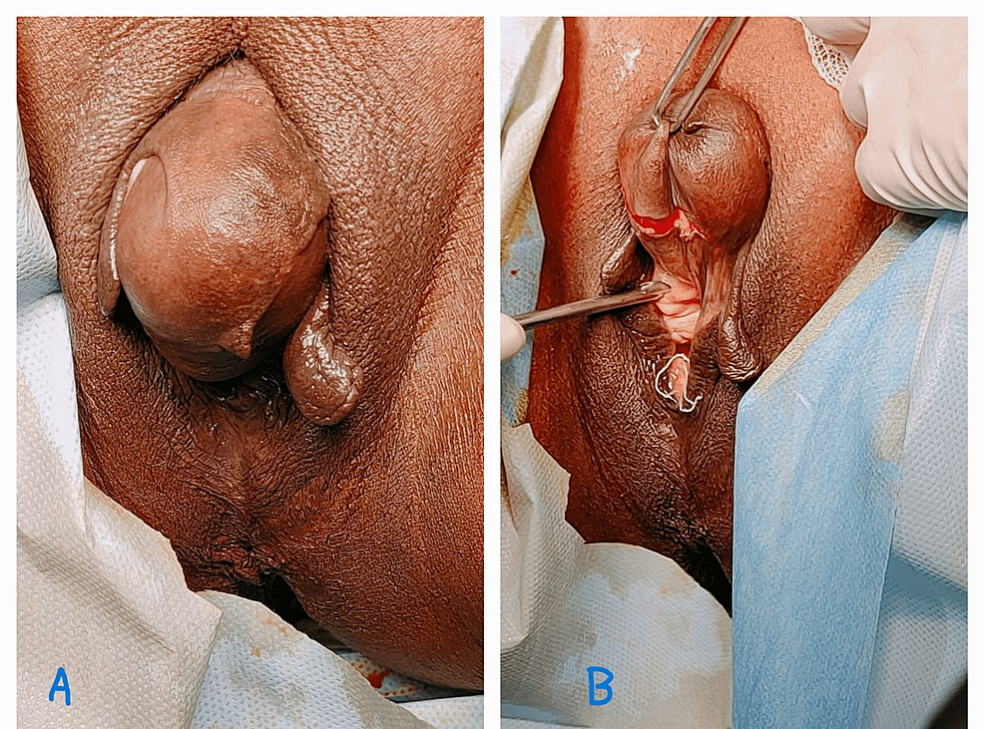
Image showing clitoral cyst before dissection A) The clitoral cyst before dissection B) Curvilinear incision given before enucleation and relation to the urethra

**Figure 2:**
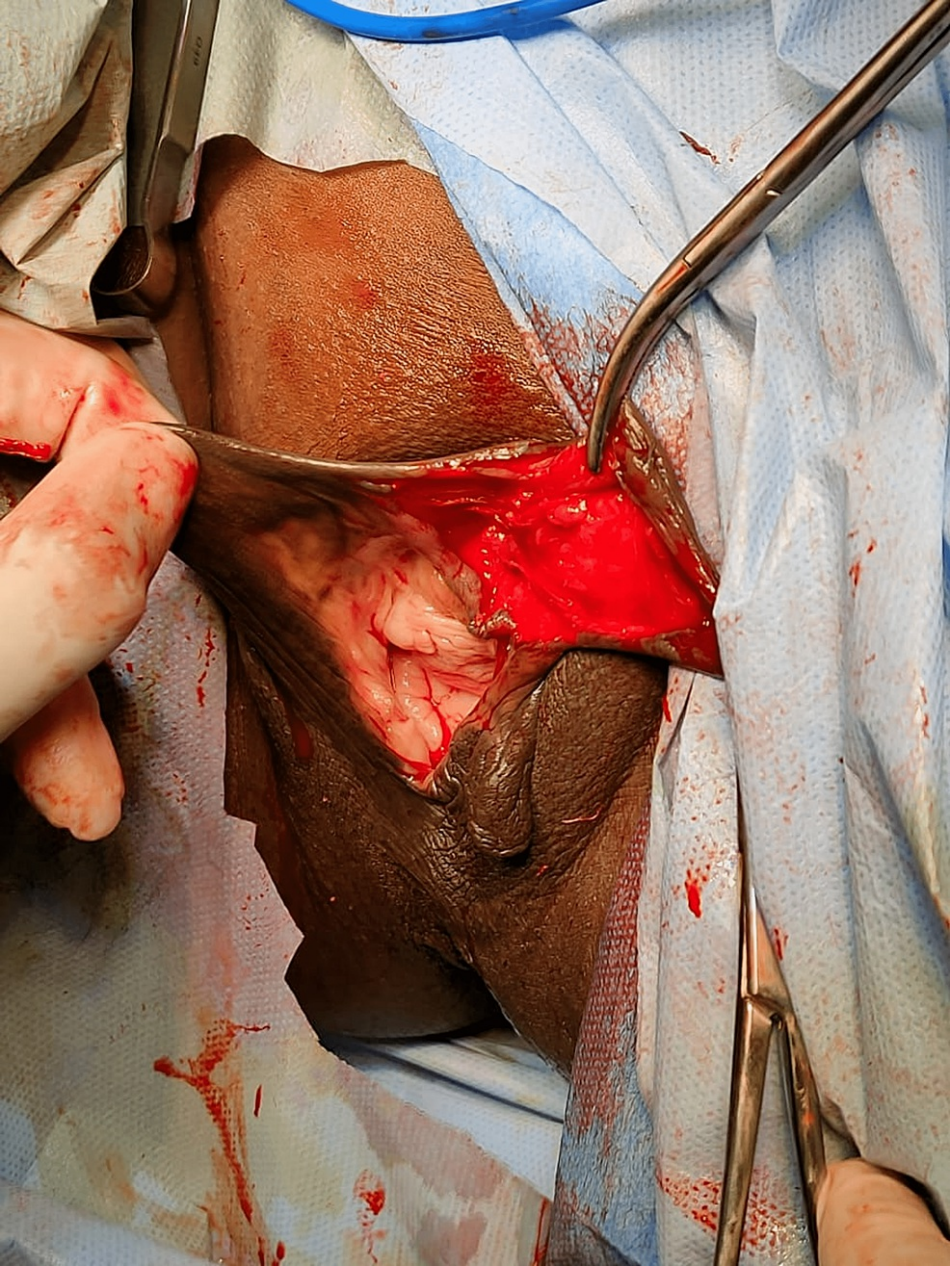
Image showing dead space after enucleation of the clitoral cyst

We present a case of epidermal inclusion cyst of the clitoris in a 43-year-old parity four female who presented to our out-patient department with complaints of swelling in the perineal region for the past 18 years, discomfort and pain during sexual intercourse for the past two to three years which increased in the past two to three months. The swelling when first noticed was initially the size of a pea, which showed very slow growth over time till it reached its present size. The onset of the swelling had no relation to delivery or trauma in the clitoral region during delivery. The swelling was first noticed by the patient four years after her last vaginal delivery. There were no urinary complaints, no history of trauma in the perineal region, no history of genital mutilation, no history of systemic complaints, and no history of hormonal intake. There was no significant obstetric, past medical, surgical, or family history. On examination, vitals were stable. Systemic examination was normal. On local examination, there was a well-circumscribed, mobile, round, soft, cystic, fluctuant, mildly tender swelling of size 6cm×4cm in the clitoral area. The skin above the swelling was pinchable. There was no hyperpigmentation or any presence of punctum. There was no local rise in temperature over the cyst. The urethra was situated very close to the swelling and appeared as if making the floor of the swelling. There were no signs of virilization. Figure 1 shows the images of the clitoral cyst before surgery and Figure 2 shows the image after the enucleation of the cyst.

**Figure 3:**
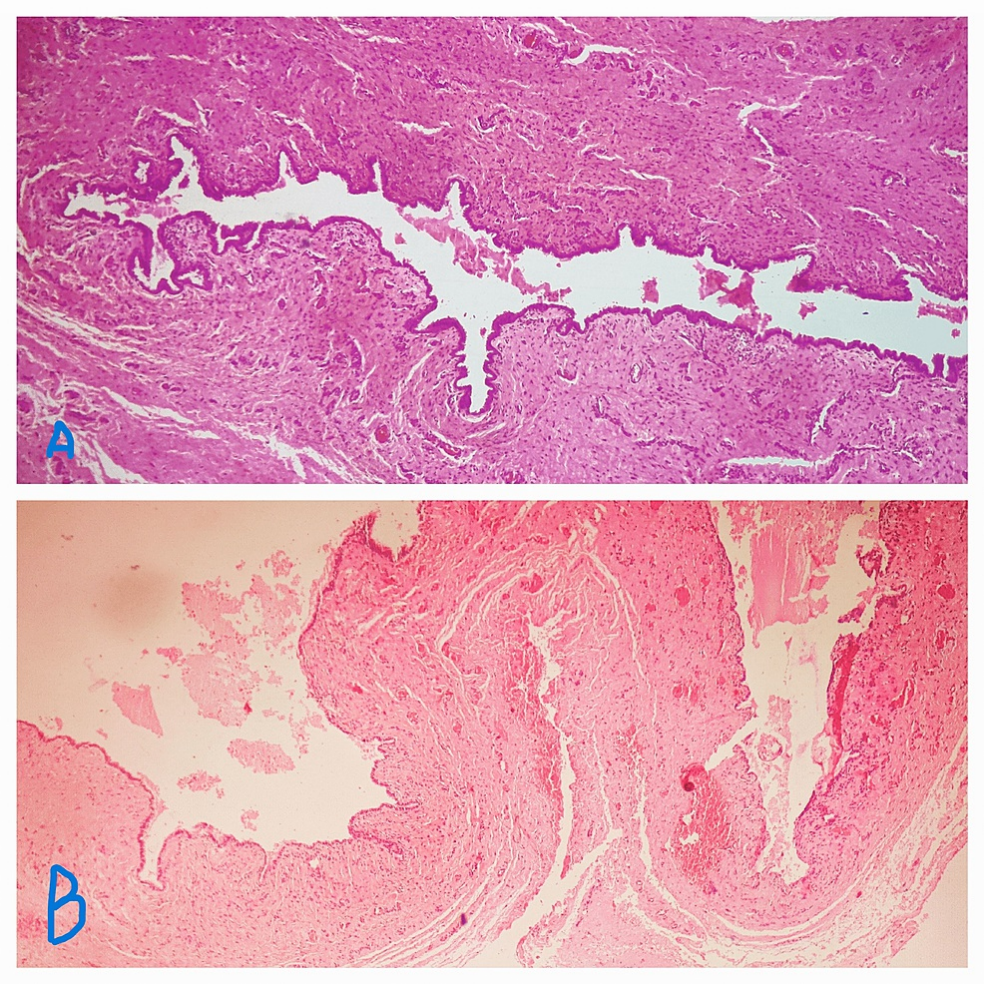
Image of histopathological examination of the clitoral epidermal inclusion cyst. Slides A and B show squamous epithelium in the cyst wall with evidence of keratinous material inside confirming the diagnosis of the epidermal inclusion cyst

Ultrasonography showed an anechoic cyst of size 6cm×4cm×3cm in the clitoral region with echogenic contents and without any vascularity. The cyst seemed to displace the urethra to the right side. However, the space for dissection between the urethra and swelling was just 3-4 mm in around 2cm length posteriorly. The features were suggestive of a clitoral cyst. After taking informed written consent, elective surgery was done and the cyst was excised under general anesthesia. A curvilinear incision was given at the mucocutaneous junction, avoiding neurovascular bundle areas of the clitoris. The urethra was kept catheterized using a metal catheter when the cyst was dissected from the posterior wall. The urethra was safely dissected away from the cyst and the cyst was enucleated. Dead space was obliterated. Hemostasis was achieved. Surgical reconstruction of the clitoris was done for cosmesis. The incision was closed with interrupted 3.0 vicryl in multiple layers. On the cut section, the cyst was filled with dirty greyish yellow material. Figure 3 shows the image of the histopathological slide examination of the clitoral epidermal inclusion cyst.

On histopathology, squamous epithelium was seen in the cyst wall with evidence of keratinous material inside confirming the diagnosis of the epidermal inclusion cyst. No sebaceous gland, sweat gland, or hair follicle was seen on histopathology thus ruling out the other differentials like a sebaceous cyst, pilar cyst, or hidradenitis. The patient had an uneventful post-operative period. On follow-up after three months, the patient was totally symptom-free with no discomfort on sexual activity and no signs of recurrence.

## Discussion

A variety of cystic tumors can occur in female external genitalia which may be vaginal, paraurethral, or clitoral [4]. Clitoral tumors, although uncommon, can be benign or malignant. Benign tumors include leiomyoma, pseudo-lymphoma, fibroma, angiokeratoma, hemangioma, hemangiopericytoma, granular cell tumor, and neurofibroma [1]. Malignant tumors reported include endodermal sinus tumor, carcinoma, sarcoma, schwannoma, lymphoma, rhabdomyosarcoma, epithelioid hemangioendothelioma, or rarely secondary metastasis [1]. Clitoral cysts usually present as swelling over the clitoris. It should be differentiated from clitoromegaly which may be due to hormonal causes. A good clinical examination should be sufficient in differentiating the clitoral cyst from clitoromegaly (uniform and symmetrical enlargement of the clitoris) [4]. Clitoral epidermoid cysts are usually common in infancy and childhood or are common after female genital mutilation or female circumcision practiced in some communities [4]. Only a few cases that could be counted on fingers have been reported that are not associated with the mutilation of female external genitalia. The case presented above is one of such few cases that was not associated with genital mutilation or circumcision and did not present in infancy or childhood. Preferred management is cyst enucleation and external genitalia reconstruction which was also done in our case. The areas of concern to be kept in mind include the impact on sexual function, cosmesis, recurrent urinary tract infection, obstetric sequelae, clitoral scarring, psychological impact, and recurrence. Our patient had no such concerns when she was examined on follow-up.

It is not uncommon to encounter clitoral epidermal inclusion cysts after a history of trauma or female genital mutilation. However, it is quite rare to see spontaneous clitoral inclusion cysts in the absence of trauma or genital mutilation. After a thorough and extensive review of the literature, we could find only ten such cases reported in the literature till date. Teague and Anglo (1996) reported a case of a clitoral cyst in a child [5]. Schmidt et al. (1999) reported an epidermal cyst of the clitoris in a 16-year-old girl as a rare cause of clitoromegaly [1]. Cetinkursun et al. (2009) also reported an epidermoid cyst causing clitoromegaly in a child [6]. Anderson-Mueller et al. (2009) reported an epidermoid cyst of the clitoris as an unusual cause of clitoromegaly in a 17-year-old female without a history of previous female circumcision [2]. Aggarwal et al. in 2010, reported an epidermoid cyst of the clitoris mimicking clitoromegaly in a 5-year-old girl [4]. Buerdeley et al. (2012) reported such a case in an 11-year-old girl [7]. Saha M (2013) reported a 5-year-old girl with clitoromegaly due to an epidermoid cyst [3]. Bhuria et al. (2014) also reported an epidermoid cyst of the clitoris in a 50-year-old multiparous female [8]. DiCarlo-Meacham et al. (2020) reported a case of a clitoral epidermal inclusion cyst in a 15-year-old girl leading to anorgasmia [9]. Lastly, Doan et al. (2021) reported a case of an infected epidermal cyst of the clitoris in an infant [10].

## Conclusions

Although rare, clitoral cysts can occasionally be seen in one’s clinical practice. It is important to determine whether the tumor is benign or malignant by histopathological examination. In the case of a benign clitoral cyst, differentiation should be done from clitoromegaly which has a whole different lot of differential diagnosis. History of trauma or mutilation should be enquired. Surgery should take into account the patient’s symptoms, cosmesis, and the impact on sexual function and psychological status. The patient should be followed up to check for recurrence.
